# Atrial fibrillation bleeding risk and prediction while treated with direct oral anticoagulants in warfarin‐naïve or warfarin‐experienced patients

**DOI:** 10.1002/clc.23887

**Published:** 2022-08-09

**Authors:** Alexander C. Perino, Jun Fan, Krishna Pundi, Susan Schmitt, Mitra Kothari, Natasha Din, Paul A. Heidenreich, Mintu P. Turakhia

**Affiliations:** ^1^ Veterans Affairs Palo Alto Health Care System Palo Alto California USA; ^2^ Department of Medicine Stanford University Stanford California USA; ^3^ Center for Digital Health Stanford University Stanford California USA

**Keywords:** atrial fibrillation, direct oral anticoagulant, discrimination, warfarin

## Abstract

**Background:**

In patients with atrial fibrillation (AF) treated with direct oral anticoagulants (DOAC), bleeding risk scores provide only modest discrimination for major or intracranial bleeding. However, warfarin experience may impact HAS‐BLED  (Hypertension, Abnormal renal/liver function, Stroke, Bleeding history or predisposition, Labile international normalized ratio, Elderly (>65 years), Drugs/alcohol concomitantly) score performance in patients evaluated for DOACs, as HAS‐BLED was derived and validated in warfarin cohorts.

**Methods:**

We performed a retrospective cohort study of patients prescribed DOAC for AF in the Veterans Health Administration between 2010 and 2017. We determined modified HAS‐BLED score discrimination and calibration for bleeding, for patients treated with DOAC, stratified by prior warfarin exposure. We also determined the association between DOAC–warfarin‐naïve status to bleeding (nonintracranial and intracranial) with DOAC–warfarin‐experienced patients as reference.

**Results:**

The DOAC analysis cohort included 100, 492 patients with AF (age [mean ± SD]: 72.9 ± 9.6 years; 1.7% female; 90.1% White), of which 26, 760 patients (26.6%) and 73, 732 patients (73.4%) were warfarin experienced or naïve, respectively. HAS‐BLED discrimination for bleeds was modest for patients treated with DOAC, regardless of prior warfarin experience (concordance statistics: 0.53–0.59). For DOAC–warfarin‐naïve patients, as compared to DOAC–warfarin‐experienced patients, adjusted risk of intracranial bleeding was lower, while risk of nonintracranial bleeding was higher ( *intracranial bleeding* propensity adjusted with inverse probability of treatment weights [IPTWs]: hazard ratio [HR]: 0.86, 95% confidence interval [CI]: 0.78–0.95, *p* = .0040) (*nonintracranial bleeding* propensity adjusted with IPTW: HR: 1.15, 95% CI: 1.11–1.19, *p* < .0001).

**Conclusion:**

Patients’ modified HAS‐BLED score at the time of DOAC initiation, regardless of prior warfarin use, provided only modest discrimination for intracranial and nonintracranial bleeds. These data argue against maintaining DOAC eligible patients on warfarin therapy regardless of modified HAS‐BLED score.

**Abbreviations**
AF: atrial fibrillation
CI: confidence interval
DOAC: direct oral anticoagulant
ICD‐10: International Statistical Classification of Diseases and Related Health Problems, tenth revision
ICD‐9: International Statistical Classification of Diseases and Related Health Problems, ninth revision
IPTW: inverse probability of treatment weights
OAC: oral anticoagulant
VA: Veterans Health Administration

## 1 INTRODUCTION

For patients with nonvalvular atrial fibrillation (AF) at increased risk of stroke, contemporary clinical practice guidelines recommend direct oral anticoagulants (DOACs) as the preferred anticoagulation strategy (Class I recommendations for all).^1^, ^2^ However, on treatment bleeding remains a chief concern for patients and clinicians when considering initiation of DOAC or transition from warfarin to DOAC in those tolerating warfarin.^3^ Bleeding risk scores, such as HAS‐BLED (Hypertension, Abnormal renal/liver function, Stroke, Bleeding history or predisposition, Labile international normalized ratio, Elderly (>65 years), Drugs/alcohol concomitantly), are commonly used in clinical practice despite (1) the lack of strong endorsement in consensus statements,^1^, ^2^ and (2) suboptimal bleeding prediction in DOAC‐treated patients studied in real‐world cohorts.^4^, ^5^, ^6^ However, estimation of bleeding risk may differ at the time of DOAC initiation based on prior warfarin exposure, due to derivation and validation of the HAS‐BLED score in warfarin cohorts,^7^, ^8^ which has not previously been explored. Notably, other bleeding risk scores, such as ORBIT (Outcomes Registry for Better Informed Treatment of Atrial Fibrillation) and ATRIA (Anticoagulation and Risk Factors in Atrial Fibrillation), have not consistently outperformed HAS‐BLED in real‐world DOAC cohorts.^6^, ^9^

Considering fragmentation of care in the United States health care system, determining prior warfarin exposure in real‐world DOAC cohorts may not always be feasible, particularly for those derived from private payer claims data.^4^, ^5^ However, the Veterans Health Administration (VA), with high rates of patient retention and satisfaction, is the largest integrated health care system in the United States. As such, patient's complete anticoagulation history can be determined. Therefore, utilizing VA data, we sought to determine the (1) association of warfarin exposure in DOAC‐treated patients to outcomes, including bleeding and death; and (2) performance of the HAS‐BLED bleeding risk score in DOAC‐treated patients with and without prior warfarin exposure.

## 2 METHODS

We performed a retrospective cohort study utilizing VA health care system data from October 1, 2010 to September 30, 2017. Linked data sets, representing the administrative and electronic health records for all VA users, included (1) the VA National Patient Care Database,^10^ (2) the VA Decision Support System national pharmacy extract,^11^ (3) the VA Fee Basis Inpatient and Outpatient data sets, (4) the VA Laboratory Decision Support System extract,^12^ (5) Medicare inpatient and outpatient institutional claims data (Part A, Part B, and carrier files),^13^ which allows for outcomes to be ascertained when veterans’ care is paid for by Medicare outside the VA health care system, and (6) the VA Vital Status File,^14^, ^15^ which contains validated combined mortality data from VA, Medicare, and Social Security Administration sources. Methods for cohort creation have been previously described in detail.^16^, ^17^, ^18^

We identified patients prescribed an OAC with a contemporaneous diagnosis of AF, which we identified using primary or secondary International Statistical Classification of Diseases and Related Health Problems, Ninth (ICD‐9) or Tenth (ICD‐10) Revision, diagnosis codes (ICD‐9: 427.3X; ICD‐10: I48.X). From patients prescribed ≥30 days of DOAC (i.e., apixaban, dabigatran, or rivaroxaban), we excluded patients (1) prescribed DOAC outside the continental United States; (2) with different DOACs prescribed on the index DOAC prescription date; (3) without an AF diagnosis within the 90 days  prior to 30 days after the index DOAC prescription date; (4) with alternate indications for anticoagulation (i.e., prior deep venous thrombosis, pulmonary embolism, or mechanical heart valve); (5) who did not establish VA care in the 4 years prior to index DOAC prescription, so warfarin use before DOAC prescription could be determined; and (6) less than 18 years of age at the time of DOAC prescription. We created a parallel warfarin cohort, using the DOAC cohort exclusion logic outlined above, from which we also excluded patients prescribed ≥30 days of DOAC prior to index warfarin prescription.

The primary predictor was the OAC treatment group, which were: (1) DOAC–warfarin naïve; 2) DOAC–warfarin experienced; and (3) warfarin. We defined warfarin experienced as a prescription for ≥30 days of warfarin in the 4 years to 14 days prior to index DOAC prescription. The warfarin and DOAC–warfarin‐experienced cohorts were not mutually exclusive. However, follow‐up time did not overlap for patients included in both cohorts. To determine the modified HAS‐BLED score, we assigned one point each for the presence of hypertension, abnormal renal function, abnormal liver function, prior stroke, prior bleeding, age ≥65 years, concurrent antiplatelet therapy, or alcohol use. We identified these component comorbidities up to 2 years preceding index OAC prescription by CPT, ICD‐9, and ICD‐10 codes. To augment the identification of abnormal renal function, we also determined if patients had an encounter at a VA dialysis clinic or a creatinine or ≥2.26 mg/dl (Supporting Information: Supplemental Table 1).^19^ Calculated HAS‐BLED scores excluded the labile international normalized ratio component because this is inapplicable across the whole cohort and cannot be obtained at baseline before treatment.

Baseline characteristics were determined using previously described methods.

The primary outcomes of interest were intracranial and nonintracranial major bleeding events, defined as an inpatient VA, VA fee basis, or Medicare encounter with an ICD‐9 or ICD‐10 bleeding diagnosis code in the primary or secondary position (Supporting Information: Supplemental Table 2). Intracranial bleeding was stratified by (1) nontraumatic or (2) traumatic, while nonintracranial bleeding was stratified by (1) gastrointestinal, (2) genitourinary, (3) respiratory tract, (4) hemarthrosis, or (5) intraocular. For encounters with bleeding diagnosis codes in both the primary and secondary positions, only the primary position was included. Death was a secondary outcome of interest.

To perform an on‐treatment analysis, we defined patients as off treatment (censored) at the time of death, intracranial or nonintracranial major bleeding event, implantation of a mechanical heart valve, prescription for a different OAC than the index OAC, and discontinuation of index OAC. We defined OAC discontinuation as a cancelation order or no OAC represcription within 30 days of the date on‐hand OAC would be estimated to run out. This estimated date was determined based on prescription date, prescribed pill count, and ideal adherence to prescribed administration frequency.

### 2.1 Statistical analysis

Differences in baseline characteristics between OAC treatment groups were assessed with the *χ* ^2^ test and two‐sample *t* test for categorical and continuous variables, respectively.

We determined the incidence rates for outcomes of interest for patients who were DOAC treated (stratified by prior warfarin experience) and warfarin treated. We also determined the associations between DOAC–warfarin‐naïve treatment and outcomes of interest, using two separate reference treatment groups: (1) DOAC–warfarin experienced and (2) warfarin. Associations were determined using univariate and multivariate Cox proportional hazards models. Multivariable models included all baseline variables as covariates.

To further evaluate associations of interest, we also performed a propensity score analysis using inverse probability of treatment weights (IPTWs). Propensity scores were calculated using logistic regression, with conditional probability of OAC treatment group based on baseline covariates (excluding baseline medications to avoid overfitting). Model fit was assessed by the Hosmer–Lemeshow goodness‐of‐fit test and concordance statistic (*C*‐statistic). Covariate weights were calculated as the inverse of the estimated propensity score for DOAC‐treated patients who were warfarin naïve and the inverse of 1 minus the estimated propensity score for DOAC‐treated patients who were warfarin experienced. Separately, we calculated covariate weights to compare DOAC–warfarin‐naive and DOAC–warfarin‐treated patients. Balance diagnostics were assessed using the standardized difference in baseline covariates before and after IPTWs. A standardized difference after IPTW < 0.1 is acceptable (Supporting Information: Supplemental Table 3).

We determined HAS‐BLED score discrimination, by OAC treatment groups, for intracranial and nonintracranial bleeding using the *C*‐statistic and Harrell *C*‐statistic. Harrell *C*‐statistic is a version of *C*‐statistic defined specifically for survival analysis.^20^ HAS‐BLED score was evaluated both as continuous and categorical variables (0–1, 2, and ≥3). *C*‐statistics range from 0.5 (no discrimination) to 1 (perfect discrimination). Receiver‐operating characteristic curves were also used to further assess discrimination. We evaluated HAS‐BLED score calibration for each OAC treatment group by plotting the major bleeding incidence rate by HAS‐BLED score in our cohort versus the original HAS‐BLED score derivation cohort.^7^, ^8^ We also determined HAS‐BLED score discrimination and calibration when major bleeding was censored at 1 year, as HAS‐BLED was originally developed to predict bleeding within 1 year of OAC initiation.

The local Institutional Review Board (Stanford, CA) and the VA Research and Development Committee (Palo Alto, CA) approved this study and waived patient consent. The last and corresponding author had full access to all study data and take responsibility for its integrity and the data analysis. All analyses were performed using SAS® software, version 9.2 (SAS Institute Inc.,Cary, NC) and STATA version 11.0 (Stata Corp, College Station, Texas).

## 3 RESULTS

**Figure 1:**
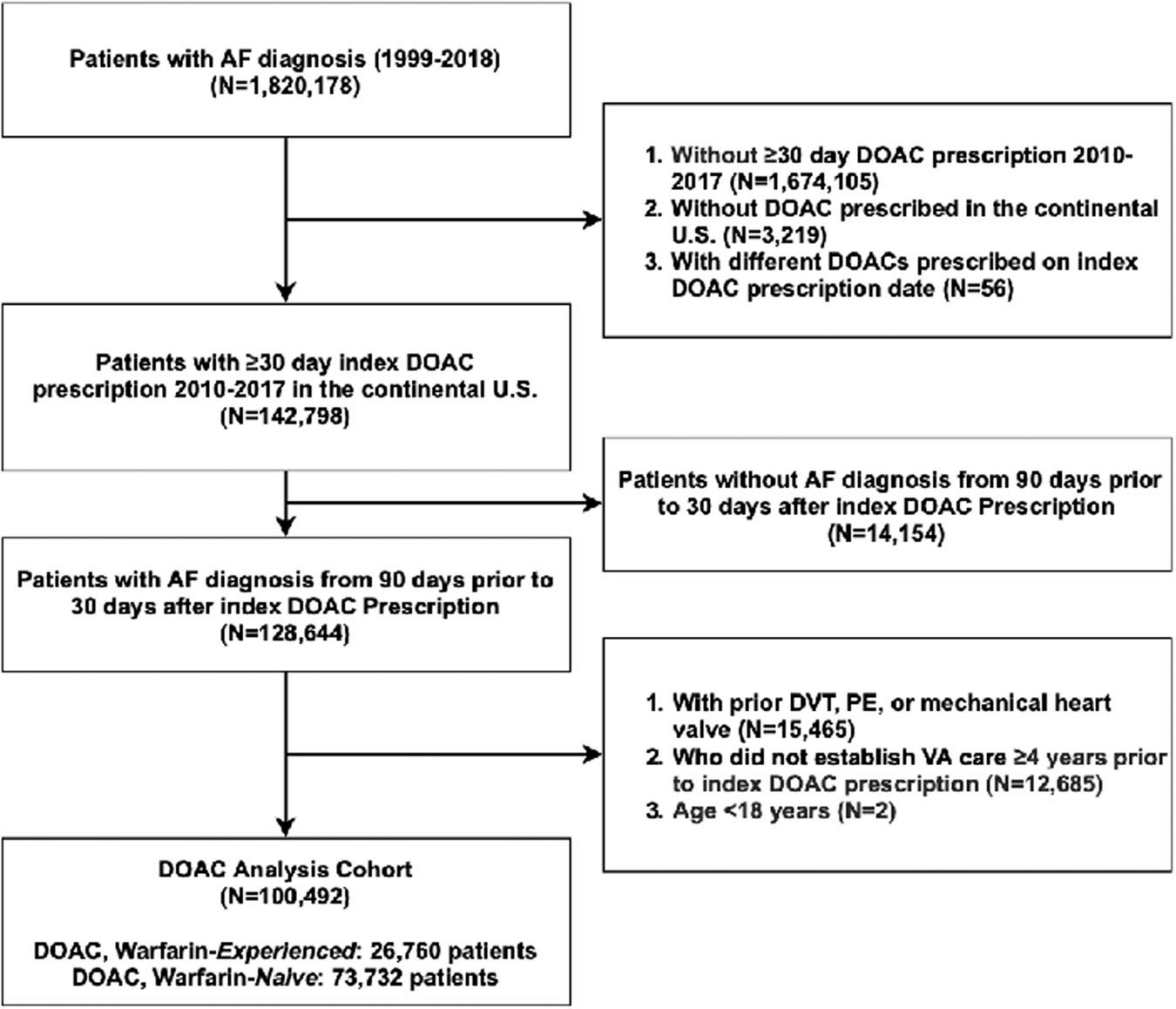
DOAC cohort selection diagram. Inclusion and exclusion criteria were used to select DOAC cohort. AF, atrial fibrillation; FY, financial year; DOAC, direct oral anticoagulant; DVT, deep vein thrombosis; PE, pulmonary embolus; US, United States; VA, Veterans Health Administration.

The DOAC analysis cohort included 100, 492 patients with AF (age: 72.9 ± 9.6 years; 1.7% female; 90.1% White). Of these, 26, 760 patients (26.6%) were warfarin experienced and 73, 732 patients (73.4%) were warfarin naïve (Figure 1). The warfarin analysis cohort included 99, 143 patients (age: 71.1 ± 9.9 years; 1.6% female; 87.6% White) (Supporting Information: Supplemental Figure 1), with 16, 367 patients (62.2% of the DOAC–warfarin‐experienced cohort) meeting inclusion criteria for both warfarin and DOAC–warfarin‐experienced cohorts. For patients in both cohorts, there was a median 1 day (25th–75th: 0–46 days) from warfarin prescription end date to index DOAC prescription date. Warfarin‐naïve patients, as compared to warfarin‐experienced patients, were older (73.6 ± 9.6 vs. 71.3 ± 9.2, *p* < .0001), had lower HAS‐BLED scores (2.4 ± 1.0 vs. 2.8 ± 1.1, *p* < .0001) and less prior bleeds (4.5% vs. 11.9%, *p* < .0001), and were less likely to have prevalent cardiovascular disease. Similar differences were observed between DOAC–warfarin‐naïve and warfarin patients, with the magnitude of the differences smaller than those observed between the DOAC cohorts (Table 1). Overall and component HAS‐BLED scores by bleed outcome and treatment group are reported in Supporting Information: Supplemental Table 4.

**Table 1:** Baseline characteristics

Demographics	Warfarin^a^ (*N* = 99 143)	DOAC–warfarin experienced^a^ (*N* = 26 760)	DOAC–warfarin naïve (*N* = 73 732)	*p* Value^b^	*p* Value^c^
Age (years)	71.1 ± 9.9	71.3 ± 9.2	73.6 ± 9.6	.0059	<.0001
Male	97 535 (98.4%)	26 291 (98.3%)	72 541 (98.4%)	.1363	.1312
Race				.0075	<.0001
White	86 810 (87.6%)	23 559 (88.0%)	66 954 (90.8%)		
Black	9463 (9.5%)	2393 (8.9%)	5037 (6.8%)		
Other/unknown	2870 (2.9%)	808 (3.0%)	1741 (2.4%)		
Comorbidities					
Coronary artery disease	34 894 (35.2%)	10 727 (40.1%)	21 900 (29.7%)	<.0001	<.0001
Chronic kidney disease	20 606 (20.8%)	8575 (32.0%)	17 553 (23.8%)	<.0001	<.0001
Diabetes	42 960 (43.3%)	12 989 (48.5%)	25 799 (35.0%)	<.0001	<.0001
Heart failure	22 542 (22.7%)	9213 (34.4%)	10 346 (14.0%)	<.0001	<.0001
Hypertension	73 839 (74.5%)	22 217 (83.0%)	49 532 (67.2%)	<.0001	<.0001
Peripheral vascular disease	7289 (7.4%)	2238 (8.4%)	3342 (4.5%)	<.0001	<.0001
Prior bleed	6185 (6.2%)	3192 (11.9%)	3295 (4.5%)	<.0001	<.0001
Prior MI	5573 (5.6%)	1264 (4.7%)	1730 (2.4%)	<.0001	<.0001
Prior stroke/TIA	9995 (10.1%)	3696 (13.8%)	4671 (6.3%)	<.0001	<.0001
Charlson Comorbidity Index	2.2 ± 1.7	2.5 ± 1.8	1.7 ± 1.5	<.0001	<.0001
CHA_2_DS_2_‐VASc score	3.1 ± 1.6	3.5 ± 1.5	2.9 ± 1.4	<.0001	<.0001
HAS‐BLED score	2.6 ± 1.2	2.8 ± 1.1	2.4 ± 1.0	<.0001	<.0001
HAS‐BLED score group				<.0001	<.0001
HAS‐BLED 0–1	13 657 (13.8%)	2483 (9.3%)	10 463 (14.2%)		
HAS‐BLED 2–4	74 371 (75.0%)	21881 (81.8%)	58 228 (79.0%)		
HAS‐BLED 5+	6094 (6.2%)	1931 (7.2%)	2528 (3.4%)		
Baseline medications					
Aspirin	27 885 (28.1%)	5501 (20.6%)	12 831 (17.4%)	<.0001	<.0001
P2Y_12_ inhibitor	34 777 (35.1%)	6611 (24.7%)	17 646 (23.9%)	<.0001	.0115
ACE‐I/ARB/ARNi	60 471 (56.5%)	16 225 (60.6%)	36 750 (49.8%)	.2813	<.0001
Diuretic	54 568 (55.0%)	14 063 (52.6%)	29 427 (39.9%)	<.0001	<.0001
Statin	67 130 (67.7%)	19 144 (71.5%)	45 770 (62.1%)	<.0001	<.0001
Rhythm control agents					
Class 1	1964 (2.0%)	891 (3.3%)	2539 (3.4%)	<.0001	.3792
Class 3	3550 (4.0%)	2073 (7.8%)	3909 (5.3%)	<.0001	<.0001
Amiodarone/dronedarone	11 973 (12.1%)	3135 (11.7%)	7542 (10.2%)	.1066	<.0001
Rate control agents					
Digoxin	13 268 (13.4%)	4049 (15.1%)	5849 (7.9%)	<.0001	<.0001
Beta‐blockers	72 878 (73.5%)	19 167 (71.6%)	43 557 (59.1%)	<.0001	<.0001
Calcium channel blockers^e^	38 964 (39.3%)	9360 (35.0%)	24 309 (33.0%)	<.0001	<.0001

*Note*: Values are represented as mean ± SD or *n* (%).

Abbreviations: ACE‐I, angiotensin‐converting enzyme inhibitors; ARB, angiotensin receptor blocker; ARNi, angiotensin receptor–neprilysin inhibitors; DOAC, direct oral anticoagulant; MI, myocardial infarction; TIA, transient ischemic attack.

^a^ Groups are not mutually exclusive, as patients may transition from warfarin to DOAC.

^b^ Differences between *warfarin* and *DOAC–warfarin‐naïve* groups were assessed with the *χ* ^2^ test and two‐sample *t* test for categorical and continuous variables, respectively.

^c^ Differences between *DOAC–warfarin‐experienced* and DOAC–warfarin‐naïve groups were assessed with the *χ* ^2^ test and two‐sample *t* test for categorical and continuous variables, respectively.

^d^ Excluding amiodarone and dronedarone.

^e^ Nondihydropyridine.

### 3.1 Outcomes for DOAC–warfarin‐naïve patients as compared to DOAC–warfarin‐experienced patients

For DOAC–warfarin‐naïve patients, as compared to DOAC–warfarin‐experienced patients, intracranial bleeding incidence rates were lower (5.2 [95% CI: 4.7–5.6] vs. 6.3 [95% CI: 5.6–7.1] per 1000 person‐years, *p* = .0101), which was the result of differences in nontraumatic intracranial bleeding (2.2 [95% CI: 2.0–2.6] vs. 3.1 [95% CI: 2.6–3.7] per 1000 person‐years, *p* = .0054). The incidence of nonintracranial bleeding and death was similar between groups (Table 2). For DOAC–warfarin‐naïve patients, as compared to DOAC–warfarin‐experienced patients, the adjusted risk of intracranial bleeding was lower, while the risk of nonintracranial bleeding was higher ( *intracranial bleeding* propensity adjusted with IPTW: hazard ratio [HR]: 0.86, 95% confidence interval [CI]: 0.78–0.95, *p* = .0040) (*nonintracranial bleeding* propensity adjusted with IPTW: HR: 1.15, 95% CI: 1.11–1.19, *p* < .0001) (Table 3).

**Table 2:** On treatment bleeding and death for DOAC (by prior warfarin experience) and warfarin cohorts

	Warfarin^a^ (*N* = 99 143)		DOAC–warfarin experienced^a^ (*N* = 26 760)		DOAC–warfarin naïve (*N* = 73 732)		*p* Value^b^	*p* Value^c^
Bleed type	*N* (%)	IR (95% CI)	*N* (%)	IR (95% CI)	*N* (%)	IR (95% CI)		
Intracranial bleed	1578 (1.6%)	8.5 (8.1–8.9)	251 (0.9%)	6.3 (5.6–7.1)	500 (0.7%)	5.2 (4.7–5.6)	<.0001	.0101
Nontraumatic	897 (0.9%)	4.8 (4.5–5.1)	125 (0.5%)	3.1 (2.6–3.7)	222 (0.3%)	2.2 (2.0–2.6)	<.0001	.0054
Traumatic	707 (0.7%)	3.8 (3.5–4.1)	128 (0.5%)	3.2 (2.7–3.8)	286 (0.4%)	2.9 (2.6–3.3)	.0002	.4162
Nonintracranial bleed	7662 (7.7%)	41.2 (40.3–42.2)	1708 (6.4%)	42.9 (40.9–45.0)	3930 (5.3%)	40.5 (39.3–41.8)	.4019	.0482
GI bleed	5667 (5.7%)	30.5 (29.7–31.3)	1233 (4.6%)	31.0 (29.3–32.8)	2928 (4.0%)	30.2 (29.1–31.3)	.6954	.4477
GU bleed	1452 (1.5%)	7.8 (7.4–8.2)	349 (1.3%)	8.8 (7.9–9.7)	713 (1.0%)	7.4 (6.8–7.9)	.1938	.0076
Respiratory tract bleed	524 (0.5%)	2.8 (2.6–3.1)	144 (0.5%)	3.6 (3.1–4.3)	312 (0.4%)	3.2 (2.9–3.6)	.0633	.2465
Hemarthrosis	128 (0.1%)	0.69 (0.58–0.82)	11 (<0.1%)	0.3 (0.2–0.5)	33 (<0.1%)	0.3 (0.2–0.5)	.0001	.5646
Intraocular bleed	6 (<0.1%)	0.03 (0.01–0.07)	1 (<0.1%)	0.03 (0.01–0.17)	2 (<0.1%)	0.02 (0.01–0.08)	.6262	.8485
Death	28 376 (28.6%)	90.5 (89.5–91.6)	3740 (13.9%)	73.6 (71.3–76.0)	7277 (9.9%)	62.6 (61.2–64.1)	<.0001	<.0001

Abbreviations: CI, confidence interval; DOAC, direct oral anticoagulant; GI, gastrointestinal; GU, genitourinary; IR, incidence rate per 1000 person‐years.

^a^ Groups are not mutually exclusive, as patients may transition from warfarin to DOAC.

^b^ IR differences between *warfarin* and *DOAC–warfarin‐naïve* groups assessed with Fisher exact test; mid *p* value reported.

^c^ IR differences between *DOAC–warfarin‐experienced* and *DOAC–warfarin‐naïve* groups assessed with Fisher exact test; mid *p* value reported.

**Table 3:** Association of DOAC prescription (by prior warfarin experience) to bleeding and death

	Unadjusted^a^		Multivariate regression^a^, ^b^		Propensity‐adjusted with IPTW^a^, ^c^	
Outcome	HR (95% CI)	*p* Value	HR (95% CI)	*p* Value	HR (95% CI)	*p* Value
DOAC–warfarin naïve versus DOAC–warfarin experienced (reference)						
Intracranial bleed	0.81 (0.69–0.94)	.0051	0.83 (0.71–0.98)	.0283	0.86 (0.78–0.95)	.0040
Nonintracranial bleed	0.93 (0.88–0.99)	.0208	1.16 (1.09–1.23)	<.0001	1.15 (1.11–1.19)	<.0001
Death	0.87 (0.84–0.91)	<.0001	1.01 (0.96–1.05)	.7775	1.01 (0.98–1.04)	.4092
DOAC–warfarin naïve versus warfarin (reference)						
Intracranial bleed	0.59 (0.53–0.65)	<.0001	0.60 (0.54–0.67)	<.0001	0.59 (0.55–0.63)	<.0001
Nonintracranial bleed	0.94 (0.90–0.98)	.0019	1.04 (0.99–1.08)	.1073	1.06 (1.03–1.09)	<.0001
Death	0.69 (0.67–0.71)	<.0001	0.75 (0.73–0.77)	<.0001	0.77 (0.76–0.79)	<.0001

Abbreviations: CI, confidence interval; DOAC, direct oral anticoagulant; HR, hazard ratio; IPTW, inverse probability of treatment weights.

^a^ Cox proportional hazards models with treatment site included as a random effect.

^b^ Multivariate model includes all baseline variables.

^c^ Conditional probability of treatment of interest based on all baseline variables, excluding medications to avoid overfitting. Covariate standardized mean differences and model fits by *C*‐statistic reported in Supporting Information:? Table 2.

### 3.2 Outcomes for DOAC–warfarin‐naïve patients as compared to warfarin

For DOAC–warfarin‐naïve patients, as compared to warfarin, incidence rates for nontraumatic and traumatic intracranial bleeding were lower (nontraumatic: 2.2 [95% CI: 2.0–2.6] vs. 4.8 [95% CI: 4.5–5.1] per 1000 person‐years, *p* < .0001; traumatic: 2.9 [95% CI: 2.6–3.3] vs. 3.8 [95% CI: 3.5–4.1] per 1000 person‐years, *p* < .0001). Incidence of death was higher for DOAC–warfarin‐naïve patients, as compared to warfarin (20.1 vs. 15.0 per 1000 person‐years, *p* < .0001) while nonintracranial bleeding was similar (Table 2). In adjusted analyses, risk of intracranial bleeding and death was lower for DOAC–warfarin‐naïve patients as compared to warfarin ( *intracranial bleeding* propensity adjusted with IPTW: HR: 0.59, 95% CI: 0.55–0.63, *p* < .0001) (*death* propensity adjusted with IPTW: HR: 0.77, 95% CI: 0.76–0.79, *p* < .0001) (Table 3).

**Figure 2:**
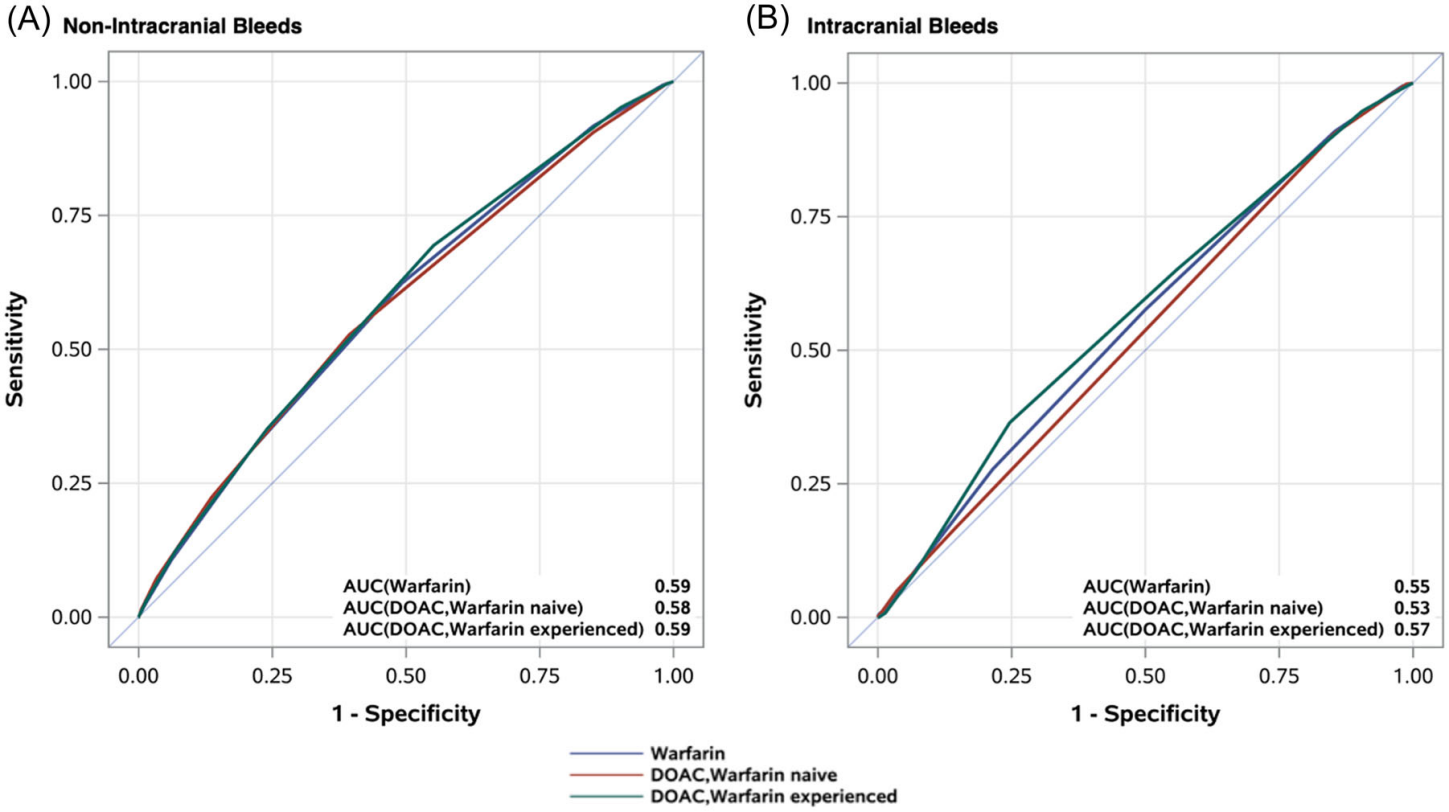
Receiver‐operating characteristic curves by bleed type for DOAC (by prior warfarin experience) and warfarin cohorts. Receiver‐operating curves for nonintracranial bleeds (A) and intracranial bleeds (B) for DOAC (warfarin naïve and warfarin experienced) and warfarin cohorts. AUC, area under the curve; DOAC, direct oral anticoagulant.

Calibration for each OAC group and the HAS‐BLED derivation cohort was similar (Supporting Information: Supplemental Figures 2 and 3). HAS‐BLED discrimination for major bleeding was modest for all treatment groups, particularly for intracranial bleeds (Table 4 and Figure 2). When major bleeding was censored at 1 year, HAS‐BLED discrimination was slightly improved numerically, as compared to when bleeding was not censored at 1 year (Supporting Information: Supplemental Table 5).

**Table 4:** HAS‐BLED discrimination by bleed type for DOAC (by prior warfarin experience) and warfarin cohorts

	Nonintracranial bleed			
	Continuous variable		Categorical variable^a^	
Cohort	*C*‐statistic (95% CI)	Harrell *C* (95% CI)	*C*‐statistic (95% CI)	Harrell *C* (95% CI)
DOAC–warfarin naïve	0.58 (0.57–0.59)	0.60 (0.59–0.61)	0.57 (0.56–0.58)	0.59 (0.58–0.60)
DOAC–warfarin experienced	0.59 (0.58–0.61)	0.63 (0.61–0.64)	0.58 (0.56–0.59)	0.60 (0.59–0.61)
Warfarin	0.59 (0.58–0.59)	0.63 (0.62–0.63)	0.57 (0.57–0.58)	0.60 (0.60–0.61)

	**Intracranial bleed**			
	**Continuous variable**		**Categorical variable** a	
**Cohort**	***C*‐statistic (95% CI)**	**Harrell *C* (95% CI)**	***C*‐statistic (95% CI)**	**Harrell *C* (95% CI)**
DOAC–warfarin naïve	0.53 (0.51–0.56)	0.56 (0.54–0.59)	0.53 (0.51–0.56)	0.56 (0.54–0.58)
DOAC–warfarin experienced	0.57 (0.54–0.60)	0.58 (0.54–0.62)	0.55 (0.52–0.58)	0.56 (0.55–0.60)
Warfarin	0.55 (0.54–0.57)	0.59 (0.57–0.61)	0.55 (0.53–0.56)	0.58 (0.57–0.59)

Abbreviations: CI, confidence interval; DOAC, direct oral anticoagulant.

^a^ HAS‐BLED categories: 0–1, 2, and ≥3.

## 4 DISCUSSION

In a large cohort of AF patients who established care within the VA healthcare system before OAC treatment, we found that modified HAS‐BLED score performance was similar for DOAC‐treated patients with and without prior warfarin exposure. Warfarin exposure before DOAC treatment was associated with more intracranial bleeding and paradoxically less nonintracranial bleeding, findings that require further exploration. These data argue against maintaining DOAC‐eligible patients on warfarin therapy, regardless of modified HAS‐BLED score.

Bleeding risk scores, including HAS‐BLED, have been shown to provide only modest discrimination for major and intracranial bleeding in DOAC‐treated patients,^4^, ^5^, ^6^ with unknown effects of prior warfarin treatment on score performance. Notably, only the ORBIT bleeding risk score was derived and validated in a DOAC cohort.^21^ Despite the HAS‐BLED score having been originally derived and validated in warfarin cohorts, our results show that HAS‐BLED score performance is not appreciably different between patients initiated on DOAC who were warfarin experienced as compared to naïve. Despite bleeding risk scores having limited use to assess the net clinical benefit of DOAC initiation in patients with AF, they may provide value for systematic identification of modifiable bleeding risk factors before OAC initiation or development of risk factors while on treatment.^22^

In seminal trials comparing DOAC to warfarin for stroke prevention in AF patients, between 50% and 60% of enrolled patients had been previously exposed to warfarin.^23^, ^24^, ^25^, ^26^ Effect modification of the primary safety endpoint, major bleeding, by prior warfarin exposure status was not observed in ARISTOTLE, ENGAGE AF‐TIMI 48, or RE‐LY.^26^, ^27^, ^28^ However, the interaction between warfarin naivety and bleeding outcomes between DOAC‐ and warfarin‐treated patients was observed in (1) ROCKET‐AF with a lower risk of major bleeding in rivaroxaban‐treated patients who were warfarin naïve as compared to warfarin experienced^29^; and (2) ARISTOTLE with a higher risk of intracranial bleeding in apixaban‐treated patients who were warfarin naïve as compared to warfarin experienced.^27^ Notably, these findings contrast with our results, which may be due to differences in demographics, comorbidities, or bleeding risk in trial versus real‐world populations. Notably, VA patients with AF are predominantly male, with larger reductions in bleeding risk for DOAC treatment, as compared to warfarin, in women.^30^ Differences in trial and real‐world outcomes may also be due to on‐treatment study designs, as opposed to intention to treat, and/or pooling of DOACs in analyses. Importantly, although evidence suggests possible differences in safety outcomes in DOAC‐treated patients based on prior warfarin exposure, they do not support warfarin maintenance in DOAC‐eligible patients.

Our study has important limitations, which include the use of a modified HAS‐BLED score partially derived from diagnostic codes. Although validated and established, diagnostic code‐derived HAS‐BLED scores may differ from clinician‐calculated HAS‐BLED scores resulting in unpredictable effects on score calibration and discrimination. Importantly, the study design did not allow for the inclusion of INR lability in our HAS‐BLED scores, which may have impacted the score's predictive ability. Although analyses adjusted for numerous baseline variables and medications, residual confounding cannot be excluded and causal inference cannot be assumed. Finally, these results may not generalize to women or outside the VA healthcare system, which utilizes pharmacist‐led anticoagulation clinics which have been associated with high rates of on‐label DOAC dosing and time in therapeutic range for warfarin‐treated patients.^31^

Patients’ modified HAS‐BLED score at the time of DOAC initiation, regardless of prior warfarin use, provided only modest discrimination for intracranial and nonintracranial bleeds. These data argue against maintaining DOAC‐eligible patients on warfarin therapy, regardless of modified HAS‐BLED score.

## CONFLICTS OF INTEREST

A. C. Perino received research support from the American Heart Association and Bristol Myers Squibb/Pfizer, and consulting for Bristol‐Myers Squibb/Pfizer. M. P. Turakhia received research support from Medtronic Inc., Abbott, Bristol Myers Squibb, American Heart Association, Biotronik, Sanofi, Pfizer, Apple Inc., Bayer, MyoKardia, Inc., Johnson & Johnson, Milestone Pharmaceuticals, JAMA. The remaining authors declare no conflict of interest.

## Supporting information

Supporting information.

## Data Availability

The data, analytic methods, and study materials will not be made available to other researchers for purposes of reproducing the results.
