# Supplementary material for: Atrial fibrillation bleeding risk and prediction while treated with direct oral anticoagulants in warfarin‐naïve or warfarin‐experienced patients
Source: Clin Cardiol. 2022 Aug 9;45(9):960–9. doi: 10.1002/clc.23887 (PMC9451662; doi:10.1002/clc.23887)
Supplement: Supplementary file 1 — Supporting information. [file CLC-45-960-s001.docx]

**SUPPLEMENTAL MATERIAL**

**TABLES**

| **Supplemental Table 1: HAS-BLED Score Component Definitions** | |  | |
| --- | --- | --- | --- |
|  | **ICD-9 Code** | **ICD-10 Code** | **CPT** |
| **Hypertension** | 401.X, 402.XX, 403.XX, 404.XX, 405.XX | I11.X, I12.X, I13.X, I15.X, I16.X |  |
| **Abnormal Renal Function** |  |  |  |
| **Dialysis patient, OR** | 996.56, 996.68, 996.73, E879.1, E870.2, E872.2, E874.2, V45.1, V45.11, V45.12, V56, V56.0, V56.1, V56.2, V56.31, V56.32, V56.8 | I95.3, R88.0, T81.502, T81.512, T81.522, T81.532, T81.592, T85.611, T85.621, T85.631, T85.691, T85.71, T82.41, T82.42, T82.43, T82.49, Y62.2, Y84.1, Z49.0, Z49.01, Z49.02, Z49.3, Z49.31, Z49.32, Z91.15, Z99.2 | A4657, 90935, 90937, 90945, 90947, 90966, 90970, 90999 |
| **Kidney transplant patient, OR** | V42.0, 55.6, 55.61, 55.69 | Z48.22, Z94.0, T86.1, T86.XX, 0TY0, 0TY1 | 00868, 50360, 50365, 50380 |
| **Creatinine >=2.26 mg/dL** |  |  |  |
| **Abnormal Liver Function** |  |  |  |
| **Viral hepatitis** | 070.0,070.1,070.2,070.3,070.4,070.5,070.6,070.7,070.9,070.XX | B15.0, B15.9,B16.0, B16.1,B16.2, B16.9,B17.0,B17.10,B17.11,B17.2,B17.8, B17.9, B18.X, B19.X, B19.XX |  |
| **Acute and subacute necrosis of liver** | 570 |  |  |
| **Chronic liver disease and cirrhosis** | 571.XX |  |  |
| **Liver abscess and sequelae of chronic liver disease** | 572.XX |  |  |
| **Other disorders of liver** | 573.X |  |  |
| **Alcohol liver disease** |  | K70.XX, K71.XX, K72.XX , K73.X, K74.XX , K75.XX, K76.X |  |
| **Prior Stroke** | 433.X1,434.X1,433.X0,434.X0,435.0,435.1,435.2,435.3,435.3,435.8, 435.9 | I63.0,I63.XX,166.XX,I67.81,I67.82,I97.81,I97.810,I97.811,I97.82,197.820,197.821,G45.X,I69.3XX |  |
| **Prior Bleeding** | 430.XX, 431.XX, 432.XX, 852.XX, 853.XX, 456.0, 456.20, 530.21, 530.7,530.82, 531.XX, 532.XX, 533.XX, 534.XX, 535.X1,562.X2, 562.X3, 578.X,719.1X | 160.0, 160.XX, 161.XX,162.XX, I69.0XX, 169.1XX,169.2XX, S06.XX, I85.01, I85.11, K22.11, K22.6, K25.XX, K26.X, K27.X, K28.X, K29.X1, K31.8X, K51.411, K55.21, K57.X, K62.5, K64.X, K66.1, K92.X, K94.XX, H05.XX, H05.XXX, H31.XX, H31.XXX, H35.6X, H35.73X, H43.1X, H44.8X, H47.02X, H60.XX, H60.XXX, M25.0X, M25.0XX, N30.01, N30.11, N30.21,N30.31,N30.41,N30.81,N30.91,N42.1, R31.0, R31.9, N99.520,N99.530,R04.0,R04.1,R04.2,R04.8,R04.89,R04.9,R58,T79.2,E07.89, E27.49,D62,D68.31,D68.311, D68.312, D68.318, D68.32 |  |
| **Age ≥ 65 years at time 0** |  |  |  |
| **Antiplatelet Therapy: Clopidogrel, Ticlopidine, Aspirin, Cilostazol, Dipyridamole, Prasugel** | Meds. identification algorithm | Meds. identification algorithm |  |
| **Alcohol** | 291.XX, 303XX, 305.X, 571.0, 571.1, 571.2, 571.3, 535.3, 535.30, 535.31, V11.3 | F10.X, G31.2,G62.1, G72.1, I42.6, K29.2X, K70.XX, K85.2, K85.20, K85.21,K85.22,K86.0, Z71.4,Z71.41, Z71.42 |  |

| **Supplemental Table 2: ICD-9/10 Codes Used to Identify Bleeding Events** | | | |
| --- | --- | --- | --- |
| **Bleed Type** | **ICD-9/10 Code Type** | **ICD-9 Code** | **ICD-10 Code** |
| **Intracranial Bleed** |  |  |  |
| **Non-traumatic** | Volume 1 and 2/CM | 430.XX, 431.XX, 432.XX | I60.XX, I61.XX, I62.XX |
| **Traumatic** | Volume 1 and 2/CM | 852.XX, 853.XX | S06.XX |
| **Non-Intracranial Bleed** |  |  |  |
| **GI Bleed** | Volume 1 and 2/CM | 456.0, 456.20, 530.21, 530.7, 530.82, 531.00, 531.01, 531.20, 531.21, 531.40, 531.41, 531.60, 531.61, 532.00, 532.01, 532.20, 532.21, 532.40, 532.41, 532.60, 532.61, 533.00, 533.01, 533.20, 533.21, 533.40, 533.41, 533.60, 533.61, 534.00, 534.01, 534.20, 534.21, 534.40, 534.41, 534.60, 534.61, 535.X1, 537.83, 537.84, 562.02, 562.12, 562.03, 562.13, 569.3, 569.85, 569.86, 578.0, 578.9 | I85.01, I85.11, K22.11, K22.6, K25.XX, K26.XX, K27.XX, K28.XX, K29.X1, K31.8X, K55.21, K57.X, K62.5, K92.X |
|  | Volume 3/PCS | 44.4X | 0DQ6, 0DQ7, 0DQ9 |
| **GU Bleed** | Volume 1 and 2/CM | 596.7, 599.71 | R31.0, R31.9 |
| **Respiratory Tract Bleed** | Volume 1 and 2/CM | 786.30, 786.39 | R04.2, R04.8, R04.89, R04.9 |
| **Hemarthrosis** | Volume 1 and 2/CM | 719.1X | M25.0X |
| **Intraocular Bleed** | Volume 1 and 2/CM | 360.43, 376.32, 377.42 | H44.8X, H05.23X, H05.23X, H47.02X |
| CM: clinical modification, GI: gastrointestinal, GU: genitourinary, ICD-9/10: International Classification of Diseases-9th/10th Revision, PCS: procedure coding system | | | |

| **Supplemental Table 3: Covariate Standardize Mean Differences Before and After IPTW** | | | | | |
| --- | --- | --- | --- | --- | --- |
| **Covariate** | **DOAC, Warfarin Naïve vs. Warfarin^1^** | |  | **DOAC, Warfarin Naïve vs. DOAC, Warfarin Experienced^2^** | |
| **Demographics** | **Before IPTW** | **After IPTW** |  | **Before IPTW** | **After IPTW** |
| **Age, years** | 0.2552 | -0.0076 |  | -0.2440 | -0.0065 |
| **Male** | -0.0005 | 0.0011 |  | 0.0107 | -0.0006 |
| **Race** |  |  |  |  |  |
| **White** | 0.1047 | -0.0083 |  | -0.0901 | -0.0058 |
| **Black** | -0.0991 | 0.0083 |  | 0.0784 | 0.0063 |
|  |  |  |  |  |  |
| **Comorbidities** |  |  |  |  |  |
| **Coronary Artery Disease** | -0.1175 | 0.0039 |  | 0.2192 | 0.0074 |
| **Chronic Kidney Disease** | 0.0727 | -0.0035 |  | 0.1844 | 0.0049 |
| **Diabetes** | -0.1715 | -0.0010 |  | 0.2774 | 0.0048 |
| **Heart Failure** | -0.2262 | 0.0129 |  | 0.4901 | 0.0017 |
| **Hypertension** | -0.1610 | 0.0036 |  | 0.3727 | 0.0062 |
| **Peripheral Vascular Disease** | -0.1195 | 0.0076 |  | 0.1564 | 0.0031 |
| **Prior Bleed** | -0.0787 | -0.0255 |  | 0.2744 | 0.1821 |
| **Prior MI** | -0.168 | 0.0113 |  | 0.1290 | 0.0031 |
| **Prior Stroke/TIA** | -0.1368 | 0.0087 |  | 0.2503 | 0.0006 |
| **Charlson Comorbidity Index** | 0.1229 | 0.0096 |  | 0.5102 | 0.0024 |
| **CHADS_2_ Score** | 0.1031 | 0.0048 |  | 0.4727 | -0.0006 |
| **CHADS_2_ Score Group** |  |  |  |  |  |
| **CHADS_2_ 0-1** | 0.1706 | -0.0039 |  | -0.3849 | -0.0024 |
| **CHADS_2_ 2-3** | -0.0886 | 0.0002 |  | 0.2094 | 0.0025 |
| **CHADS_2_ 4-6** | -0.1486 | 0.0067 |  | 0.2670 | -0.0004 |
| **CHA_2_DS_2_-VASc Score** | 0.0803 | 0.0112 |  | 0.4078 | 0.0016 |
| **HAS-BLED Score** | -0.1876 | -0.0069 |  | 0.3419 | 0.0991 |
| Guidelines suggest 0.1 or 0.25 as acceptable cutoff for standardized mean difference. DOAC: direct oral anticoagulant, IPTW: inverse probability of treatment weights, MI: myocardial infarction, TIA: transient ischemic attack.  ^1^Model fit assessed by C statistic (P=0.68) ^2^Model fit assessed by C statistic (P=0.71) | | | | | |

| **Supplemental Table 4: HAS-BLED Score and Components by Bleed Outcome and OAC Treatment Group** | | | | | | | | | | | |
| --- | --- | --- | --- | --- | --- | --- | --- | --- | --- | --- | --- |
|  | **No Bleed** | | |  | **Non-Intracranial Bleed** | | |  | **Intracranial Bleed** | | |
|  | **Warfarin N=85,367** | **DOAC,  Warfarin Experienced N=24,390** | **DOAC,  Warfarin  Naïve  N=66,955** |  | **Warfarin N=7,283** | **DOAC,  Warfarin Experienced N=1,658** | **DOAC,  Warfarin Naïve N=3,780** |  | **Warfarin N=1,472** | **DOAC,  Warfarin Experienced N=247** | **DOAC,  Warfarin Naïve N=484** |
| **HAS-BLED Score** | 2.6 ± 1.2 | 2.8 ± 1.1 | 2.4 ± 1.0 |  | 3.0 ± 1.2 | 3.2 ± 1.2 | 2.7 ± 1.1 |  | 2.9 ± 1.2 | 3.1± 1.1 | 2.6 ± 1.0 |
| **HAS-BLED Components** |  |  |  |  |  |  |  |  |  |  |  |
| **Hypertension** | 75,028 (87.9%) | 22,601 (92.7%) | 56,912 (85.0%) |  | 6,680 (91.7%) | 1,590 (95.9%) | 3,363 (89.0%) |  | 1,336 (90.8%) | 233 (94.3%) | 425 (87.8%) |
| **Abnormal Renal Function** | 4,842 (5.1%) | 410 (1.7%) | 885 (1.3%) |  | 699 (9.6%) | 57 (3.4%) | 76 (2.0%) |  | 138 (9.4%) | 3 (1.2%) | 8 (1.7%) |
| **Abnormal Liver Function** | 6,395 (7.5%) | 2,065 (8.5%) | 3,637 (5.4%) |  | 645 (8.9%) | 173 (10.4%) | 243 (6.4%) |  | 109 (7.4%) | 21 (8.5%) | 18 (3.7%) |
| **Prior Stroke** | 15,834 (18.6%) | 5,711 (23.4%) | 9,618 (14.4%) |  | 1,718 (23.6%) | 475 (28.7%) | 762 (20.2%) |  | 378 (25.7%) | 89 (36.0%) | 109 (22.5%) |
| **Prior Bleeding** | 16,513 (19.3%) | 7,855 (32.2%) | 10,349 (15.5%) |  | 2,022 (27.8%) | 721 (43.5%) | 921 (24.4%) |  | 328 (22.3%) | 93 (37.7%) | 61 (12.6%) |
| **Age ≥ 65 years** | 62,890 (73.7%) | 19,433 (79.7%) | 57,263 (85.5%) |  | 5,716 (78.5%) | 1,382 (83.4%) | 3,358 (88.8%) |  | 1,203 (81.7%) | 208 (84.2%) | 451 (93.2%) |
| **Antiplatelet Therapy** | 29,645 (34.7%) | 5,869 (24.1%) | 15,908 (23.8%) |  | 3,164 (43.4%) | 554 (33.4%) | 1,206 (31.9%) |  | 559 (38.0%) | 67 (27.1%) | 124 (25.6%) |
| **Alcohol Use** | 10,763 (12.6%) | 3,553 (14.6%) | 6,584 (9.8%) |  | 998 (13.7%) | 273 (16.5%) | 426 (11.3%) |  | 159 (10.8%) | 41 (16.6%) | 43 (8.9%) |

| **Supplemental Table 5: HAS-BLED Discrimination by Bleed Type within 1 Year for DOAC (by prior warfarin experience) and Warfarin Cohorts** | | | | |
| --- | --- | --- | --- | --- |
|  | **Non-Intracranial Bleed** | | | |
|  | **Continuous Variable** | | **Categorical Variable^1^** | |
| **Cohort** | **C-Statistic (95% CI)** | **Harrell C (95% CI)** | **C-Statistic (95% CI)** | **Harrell C (95% CI)** |
| DOAC, Warfarin-Naïve | 0.60 (0.58-0.61) | 0.61 (0.60-0.62) | 0.59 (0.58-0.60) | 0.59 (0.58-0.60) |
| DOAC, Warfarin-Experienced | 0.62 (0.60-0.64) | 0.63 (0.61-0.65) | 0.59 (0.58-0.61) | 0.60 (0.59-0.61) |
| Warfarin | 0.62 (0.61-0.63) | 0.61 (0.60-0.62) | 0.60 (0.59-0.60) | 0.60 (0.58-0.62) |
|  | **Intracranial Bleed** | | | |
|  | **Continuous Variable** | | **Categorical Variable^1^** | |
| **Cohort** | **C-Statistic (95% CI)** | **Harrell C (95% CI)** | **C-Statistic (95% CI)** | **Harrell C (95% CI)** |
| DOAC, Warfarin-Naïve | 0.56 (0.53-0.59) | 0.57 (0.54-0.60) | 0.55 (0.52-0.58) | 0.56 (0.53-0.59) |
| DOAC, Warfarin-Experienced | 0.57 (0.52-0.61) | 0.57 (0.52-0.62) | 0.55 (0.51-0.59) | 0.55 (0.51-0.59) |
| Warfarin | 0.58 (0.56-0.60) | 0.58 (0.56-0.60) | 0.57 (0.55-0.59) | 0.61 (0.60-0.62) |
| CI: confidence interval, DOAC: direct oral anticoagulant  ^1^HAS-BLED categories: 0-1, 2, ≥3 | | | | |

**FIGURES**

**Supplemental Figure 1: Warfarin Cohort Selection Diagram**


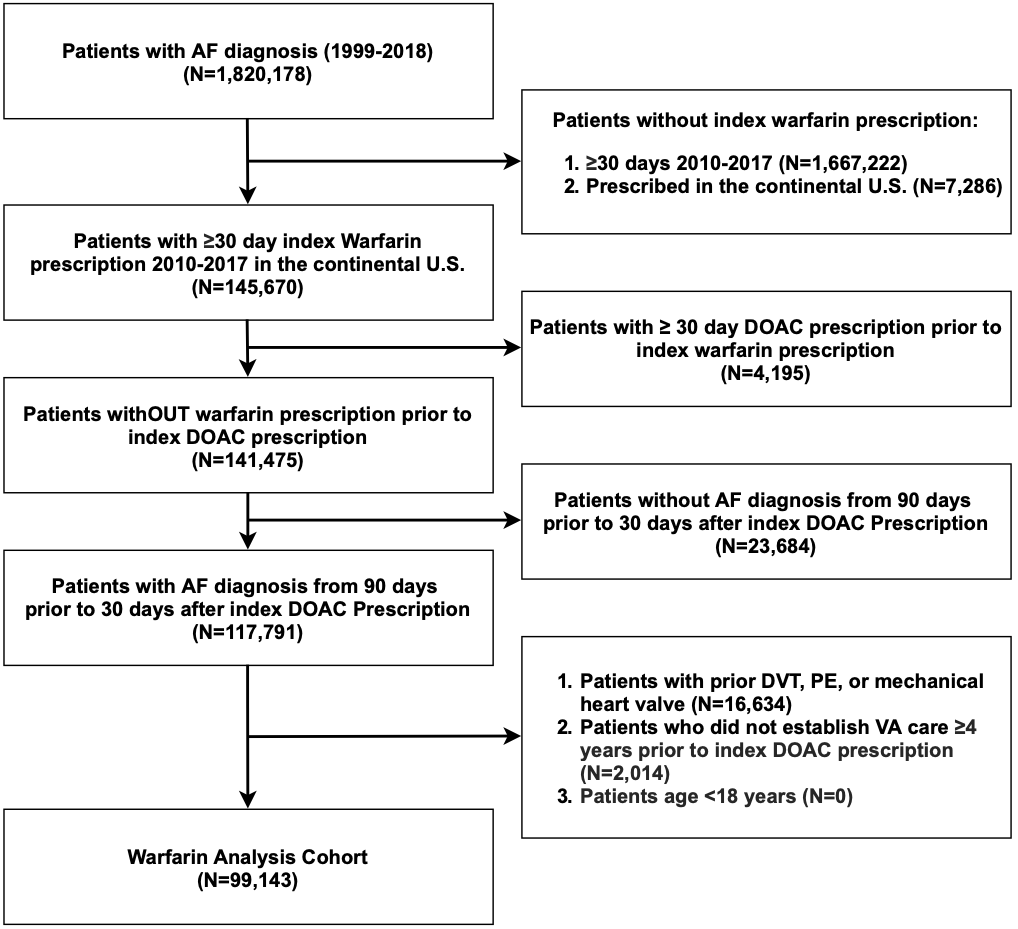


Inclusion and exclusion criteria used to select warfarin cohort. AF: atrial fibrillation, FY: financial year, DOAC: direct oral anticoagulant, DVT: deep vein thrombosis, PE: pulmonary embolus, U.S.: United States. VA: Veterans Health Administration.

**Supplemental Figure 2: Calibration Plots for Major Bleeding for DOAC (by prior warfarin experience) and Warfarin Cohorts**

Major bleeding incidence rate by HAS-BLED score in our warfarin cohort (panel A), DOAC – Warfarin Experienced cohort (panel B), and DOAC – Warfarin Naïve cohort (panel C) versus major bleeding incidence rate by HAS-BLED score in the original HAS-BLED score deriviation cohort (x-axis). DOAC: direct oral anticoagulant.

**Supplemental Figure 3: Calibration Plots for Major Bleeding within 1 Year for DOAC (by prior warfarin experience) and Warfarin Cohorts**

Major bleeding incidence rate within 1 year by HAS-BLED score in our warfarin cohort (panel A), DOAC – Warfarin Experienced cohort (panel B), and DOAC – Warfarin Naïve cohort (panel C) versus major bleeding incidence rate by HAS-BLED score in the original HAS-BLED score deriviation cohort (x-axis). DOAC: direct oral anticoagulant.
